# “Finding my way in a maze while the clock is ticking”: The daily life challenges of adolescents and young adults with an uncertain or poor cancer prognosis

**DOI:** 10.3389/fonc.2022.994934

**Published:** 2022-11-15

**Authors:** Vivian W. G. Burgers, Martin J. van den Bent, Linda Dirven, Roy I. Lalisang, Jacqueline M. Tromp, Annette Compter, Mathilde Kouwenhoven, Monique E. M. M. Bos, Adrianus de Langen, Milou J. P. Reuvers, Suzanne A. Franssen, Simone A. M. M. Frissen, Niels C. G. L. Harthoorn, Annemiek Dickhout, Marloes J. Noordhoek, Winette T. A. van der Graaf, Olga Husson

**Affiliations:** ^1^ Division of Psychosocial Research and Epidemiology, Netherlands Cancer Institute, Amsterdam, Netherlands; ^2^ Department of Medical Oncology, Netherlands Cancer Institute-Antoni van Leeuwenhoek, Amsterdam, Netherlands; ^3^ Department of Neurology, Erasmus University Medical Center, Rotterdam, Netherlands; ^4^ Department of Neurology, Leiden University Medical Centre, Leiden, Netherlands; ^5^ Department of Neurology, Haaglanden Medical Center, The Hague, Netherlands; ^6^ Division Medical Oncology, Internal Medicine, Maastricht University Medical Center, Maastricht, Netherlands; ^7^ GROW-School of Oncology and Developmental Biology, Maastricht University Medical Center, Maastricht, Netherlands; ^8^ Department of Medical Oncology, Amsterdam University Medical Centers, Amsterdam, Netherlands; ^9^ Department of Neurology, Amsterdam University Medical Centers, Vrije Universiteit, Amsterdam, Netherlands; ^10^ Brain Tumor Center Amsterdam, Cancer Center Amsterdam, Amsterdam University Medical Centers (UMC), Vrije Universiteit Amsterdam, Amsterdam, Netherlands; ^11^ Department of Medical Oncology, Erasmus Medical Center (MC) Cancer Institute, Erasmus University Medical Center, Rotterdam, Netherlands; ^12^ Department of Thoracic Oncology, Netherlands Cancer Institute-Antoni van Leeuwenhoek, Amsterdam, Netherlands; ^13^ Adolescent and Young Adult (AYA) Research Partner, Amsterdam, Netherlands; ^14^ Department of Surgical Oncology, Erasmus Medical Center (MC) Cancer Institute, Erasmus University Medical Center, Rotterdam, Netherlands; ^15^ Division of Clinical Studies, Institute of Cancer Research, London, United Kingdom

**Keywords:** adolescents and young adults, poor or uncertain cancer prognosis, psychosocial challenges, psychological therapy, qualitative research

## Abstract

**Introduction:**

Increasingly more adolescent and young adult (AYA, aged 18–39 years) patients with an uncertain and/or poor cancer prognosis (UPCP) are gaining life-years because of novel treatments or refinement of established therapies, and sometimes even face the prospect of long-term disease control. This study aims to examine the challenges of AYAs with a UPCP in daily life to inform the development of AYA care programs.

**Methods:**

Semi-structured in-depth interviews were conducted among AYAs with a UPCP. Since we expected differences in experiences between three AYA subgroups, we interviewed patients of these subgroups (1): traditional survivors (2), low-grade glioma survivors, and (3) new survivors. Interviews were analyzed using elements of grounded theory. AYA patients were actively involved as research partners.

**Results:**

In total 46 AYAs with UPCP participated and shared their challenges in daily life. They were on average 33.4 years old (age range 23–44) and most of them were women (63%). The most common tumor types were low-grade gliomas (16), sarcomas (7), breast cancers (6), and lung cancers (6). We identified five primary themes: (1) feeling inferior to previous self and others (e.g. feeling useless, who wants me in a relationship), (2) feeling of being alone (e.g. lonely thoughts, nobody really gets me), (3) ongoing confrontation (e.g. it is always there, own decline), (4) grief about life (e.g. grief about life I did not get, grief about old life), and (5) loss of control over the future (e.g. not able to make future plans, waiting for growth). Although all of the challenges were identified in the three AYA subgroups, the perceived intensity of the challenges differed slightly between the subgroups.

**Discussion:**

AYAs living with a UPCP experience challenges associated to their sense of altered identity, their position in the social network, and the future uncertainties. This study highlights the importance to recognize and acknowledge the unique challenges of this group. To provide age-specific care, it is important to embed acceptance and commitment therapy and AYA peer support within the healthcare system and other care programs to support AYAs to live well with their disease.

## Introduction

An important but often overlooked group of cancer patients are adolescents and young adults (AYA; 15–39 years of age) with an uncertain or poor cancer prognosis (UPCP). Up to now, most psychosocial research studies in this age group have been focused on patients with a curative intent, and more recently, attention is paid on those in the end-of-life phase (1, 2). However, due to advances in treatments, a growing number of AYAs with a UPCP are living longer with cancer (3, 4). We recently defined AYAs with a UPCP as those with advanced cancer for which there is no reasonable hope of cure, indicating that they will die prematurely from cancer, but have no immediate threat of death (5). Patients with terminal illness, defined as those with a life expectancy less than 3–6 months and poor performance status, do not belong to this group (5, 6). Patients with a UPCP were classified into three groups based on received treatment and prognosis, i.e. those (1) treated with standard established treatments (e.g. chemotherapy; traditional survivors with a life expectancy of 1–5 years) (2), undergoing novel treatment (s) (e.g. targeted therapy and immunotherapy; new survivors with uncertain prognosis), or (3) with a low-grade glioma who are living with the knowledge that tumor progression inevitably occurs and likely will be lethal (5).

The diagnosis of advanced cancer in adolescence or young adulthood can bring one’s life and the achievement of milestones to an abrupt halt. The attention of an AYA has to shift to cancer-related challenges including management of side effects, dealing with the threat of death and the future uncertainties; instead of developing and realizing relationships, careers, and achieving future goals (7–9). AYAs struggle with lost opportunities to participate in a long, healthy, and meaningful life, while most of them do not yet have the coping skills to deal with this situation (10). These age-related challenges make AYAs with a UPCP a vulnerable group. However, these challenges have not yet been well studied since this patient group is often “absorbed” in the large group of AYAs treated with curative intent with an overall good prognosis, in the overall adult cancer population or in the group of patients near the end of life (11). The limited qualitative research on AYAs with advanced or metastatic cancer report that AYAs experience a sense of isolation from healthy peers, AYA cancer patients with curative treatment goals, and older patients (12). The loss of control over their life and the psychological challenge of anticipatory grief over the life that has not yet been lived might be difficult for AYAs to process (10, 11, 13). Due to the prognostic uncertainty, AYAs are constantly balancing between hope and fear amidst uncertain treatment outcomes (14).

According to a meta-review study of Garcia et al., patients living with advanced cancer in general experience situations or events that involve suffering (e.g. physical symptoms, spiritual and psychological suffering) (15). Patients with advanced disease try to reduce this suffering and find new meaning in life through coping mechanisms, such as adapting to change and keeping hope. Garcia et al. identified the common desire of patients to preserve or reclaim normalcy by engaging in usual daily activities. However, this can be difficult to pursue due to physical, psychosocial, and emotional challenges (15–19). Challenges for AYA with a UPCP might be even more profound due to their unique and uncertain disease situation while being in a vulnerable developmental life phase. Therefore, the aims of this study were to examine more specifically the psychosocial challenges in daily life of the growing group of AYAs with a UPCP.

## Methods

### Study design

A qualitative study was conducted. Elements of the Grounded Theory by Corbin and Strauss were used in combination with a constructivist philosophical perspective (20). This perspective implies the acknowledgement of (1) the existence of multiple complex realities that relate to real events, (2) differences in participants’ responses to events, and (3) that the construction of theories is dependent of the researchers (20–22). This approach was chosen because of the ability to understand the actions of individuals in a context of their unique life-stage and diagnosis while facing problems or specific social situations. To ensure good quality of our study, the ‘Consolidation Criteria for Reporting Qualitative Studies’ (COREQ) guidelines were followed (23) (**Supplementary Material 1**).

### Participants

Patients were recruited in eight University Medical Centers in the Netherlands, the Netherlands Cancer Institute, and one large nonacademic teaching hospital (Haaglanden Medical Center). Eligible patients included those (1) diagnosed with any type of advanced cancer for the first time between 18 and 39 year of age, for which there is no reasonable hope of cure, indicating that the patient will die prematurely due to cancer, and (2) able to speak and understand Dutch. The age range between 18 and 39 years was chosen because this is the age definition of AYA cancer patients in the Netherlands. Patients with terminal illness with a life expectancy of 3–6 months or less and a poor performance status were excluded. This decision was based on the prognostic estimates of the treating clinician *via* a surprise question (“Would you be surprised if this patient died within the next 6 months?”). Patients with significant cognitive problems, who were not able to complete an interview as determined by the treating clinician, were also excluded.

Potential patients meeting the eligibility criteria were identified and informed by their treating clinician *via* telephone or face-to-face in clinic. After obtaining permission, the researcher contacted the patient to provide further information and obtained written informed consent. Since we expected differences in experiences between the three AYA subgroups described above (traditional survivors, new survivors, low-grade glioma survivors), we aimed to interview at least 16 patients per distinct subgroup to ensure a heterogeneous sample. The research team (VB, OH, WvdG) divided patients in the three subgroups based on information from the treating clinician regarding the received treatment and prognosis (5). We applied a purposive sampling strategy, aiming to achieve good representation of sex/gender, age, and tumor types within each subgroup. The minimum of 16 patients per subgroup was a starting point for the recruitment phase to cover the full spectrum of the diverse group of AYAs with a UPCP. Additionally, we included five eligible AYA patients (divided in the three subgroups) in a focus group, which is described in more detail in the next section. Data collection stopped when saturation was reached, next to the minimum predefined sample size, and the researcher had a clear overview of the challenges of AYAs with a UPCP experienced in daily life. Ethical approval was given by the Institutional Review Board of The Netherlands Cancer Institute (IRBd20-205). In the other participating centers, this study was approved by their ethical committees according to local regulations.

### Procedure

Interviews were conducted between April 2021 and April 2022 during the COVID-19 pandemic. A trained female psychologist and qualitative researcher (VB) conducted the semi-structured in-depth interviews *via* video calls. An interview guide with open questions and probes was created based on literature. This interview guide was drafted in collaboration with experienced researchers (WvdG, OH) and AYA patients who are actively involved as research partners in this study (AD, NH, MN, SF, SF). In order to optimize the transparent reporting of the active involvement of patients as research partners in this study, the GRIPP2-SF checklist was followed and completed (24) (**Supplementary Material 2**). The AYA research partners reviewed and adapted the interview guide for relevance, comprehensiveness, word use, and level of confrontation. The interview guide was pilot-tested and minor adjustments were done twice during the data collection to obtain the most optimal information (**Table 1**). Interviews were audio-recorded and lasted 84 min on average (range: 49–122). Notes and summaries were made to provide context for analysis. Each participant was called a week after the interview to evaluate their experience with the interview and to provide an option to share additional relevant information that was not covered during the interview.

**Table 1:** Interview topic guide.

Questions	Probes
Could you please tell me how your disease (or preferred term by AYA) has affected your life?	Could you please tell me how your disease (or preferred term by AYA) has affected your daily life?
	Could you please tell me how your disease (or preferred term by AYA) has affected important life choices? (e.g. study, job, children)
	Could you please take me through a specific case which describes your experience in the best way?
	Can you give an example that visualizes the challenges you came across when dealing with your disease?
	Do you think the impact would have been different when you know for sure that you will be cured?
Could you please tell me how your disease has affected your relationship with your loved ones?	Could you please give me an example that showcases this well?
	Who of you is changed? Could you please elaborate?
How do you experience the support you receive?	Could you please elaborate? (from who, how and what frequency do you experience support)
	What kind of advice would you give to your support system?
	What is the best way to support you right now?
	Do you miss or have missed some kind of support?
What are your thoughts about the future?	How is your future perspective changed after your diagnosis? And has this changed over time?
	What concerns you the most at the moment? What are you afraid of?
	Do you already have taken action regarding your future, which you never would have done on this point in your life if you were not diagnosed with this disease?
	What are your needs regarding communication about the future?

The preliminary results of the study were shared with the AYA research partners and discussed during focus groups with AYA research partners. The focus group aimed to check for interpretation and correctness of a patients’ point of view. Since the focus group discussions gave also additional insights into the daily challenges of AYAs with a UPCP, we decided to incorporate these findings into the main results of this study. The AYA research partners all provided informed consent to be included as participants in this study. In total, five focus groups were conducted by VB, all were audio-recorded and lasted 67 min on average (range: 53–90).

In addition to these interviews and focus groups, patients completed a short case report form (CRF) on their sociodemographic and medical characteristics including; age, gender, ethnical and religious background, level of education, employment status, living and care arrangements, age on diagnosis, and comorbidity using Charlson Index. Clinical data, including primary diagnosis, date of diagnosis, stage of disease, and type of treatment (if any) were obtained from the treating clinician after patients gave permission for requesting medical data.

### Data analysis

Interviews and focus groups were transcribed verbatim and the transcripts were analyzed by VB using elements of the grounded theory by Corbin and Strauss (20). Analysis started immediately after the first interview. A cyclic process was applied of interviewing, visualizing the interview, analyzing, and reflection by memo writing, leading to new questions and more interviews. Analysis started with open coding and axial coding with the help of NVivo (25). For the first 15 interviews (five of each subgroup), another independent researcher (MR) repeated this process, in order to identify and discuss different perspectives on the same data. In case of disagreement, the remaining codes were discussed with the AYA research partners and the research team. In the meetings with the AYA research patients and research team, everyone was equal in the decision-making process. Open coding and axial coding were done concurrently. Axial coding was done with the help of “the paradigm’ of Corbin and Strauss, an analytical tool to structure around a category in terms of conditions, actions–interactions, and consequences (20). These steps were followed for each unique interview in which each new interview was compared with the data of the other interviews. VB wrote memos, used strategies like asking questions, constant comparison, and finding the negative case(s) to understand the essence of what was being said: the underlying issue of the challenges AYAs experienced. After analyzing half and after analyzing all of the interviews, the codes were checked by the AYA research partners as a form of member checking. The focus groups were analyzed the same way as the interviews and the interpretation of the results were checked by the AYA research partners themselves. With selective coding, all the data came together to construct a plausible explanatory framework about the challenges of living with a UPCP at AYA age. This general framework was checked for misinterpretation or gaps in logic by the research team and AYA research partners, resulting in minor adjustments. Data collection stopped when conceptual saturation was reached (20).

## Results

In total, 64 patients were invited, of whom 46 (72%) were actually interviewed. Eleven patients declined due to illness, four patients were too busy or did not want to focus on their disease, and three patients did not respond. Due to a combination of reaching conceptual saturation and repeated illness of the last patients that needed to be included, we ended up with 15 patients in two subgroups and 16 in the low-grade glioma subgroup. The mean age of AYAs at time of the interview was 33.4 years (**Table 2**). The majority of the interview and focus group participants were women, had a Dutch ethnicity, were married or had a partner and did not have children (**Table 2**). A range of tumor types were included, most commonly (low-grade) gliomas, followed by sarcomas, breast cancers, and lung cancers.

**Table 2:** Demographics AYA respondents and focus group of AYA research partners.

Characteristics	Total AYAs^a^ N (%)	Traditional Survivors N (%)	New Survivors N (%)	LGG survivors N (%)	Focus group N (%)
Age at initial diagnosis, years					
Range	23–39	23–37	22–38	22–39	20–38
Mean (SD)	29.6 (4.8)	28.93 (3.9)	30.7 (4.9)	29.8 (5.7)	27.6 (6.6)
Current age, years					
Range	23-44	24-44	23-41	23-43	23-38
Mean (SD)	33.4 (6.3)	33.0 (5.6)	34.2 (4.8)	33.2 (5.6)	31.8 (5.5)
Gender					
Woman	29 (63.0)	12 (80.0)	10 (66.7)	7 (43.8)	4 (80.0)
Man	17 (37.0)	3 (20.0)	5 (33.3)	9 (56.3)	1 (20.0)
Ethnicity					
Caucasian	46 (100)	15 (100)	15 (100)	16 (100)	5 (100)
Religion					
No	38 (82.6)	13 (86.7)	13 (86.7)	12 (75.0)	5 (100)
Yes^b^	8 (17.4)	2 (13.3)	2 (13.3)	4 (25.0)	0 (0.0)
Marital status					
Married or partnered	38 (82.6)	11 (73.3)	13 (86.7)	14 (87.5)	3 (60.0)
Single	8 (17.4)	4 (26.7)	2 (13.3)	2 (12.5)	2 (40.0)
Living situation					
Living alone	7 (15.2)	3 (20.0)	3 (20.0)	1 (6.3)	1 (20.0)
Living with partner	16 (34.8)	6 (40.0)	4 (26.7)	6 (37.5)	1 (20.0)
Living with partner and children	16 (34.8)	3 (20.0)	5 (33.3)	8 (50.0)	2 (40.0)
Living with children	3 (6.5)	2 (13.3)	1 (6.7)	0 (0.0)	0 (0.0)
Living with parents	2 (4.3)	1 (6.7)	0 (0.0)	1 (6.3)	0 (0.0)
Other^c^	2 (4.3)	0 (0.0)	2 (13.3)	0 (0.0)	1 (20.0)
Children					
Yes	19 (41.3)	5 (33.3)	6 (40.0)	8 (50.0)	2 (40.0)
No	27 (58.7)	10 (66.7)	8 (60.0)	8 (50.0)	3 (60.0)
Highest achieved level of education					
Secondary education or less	4 (8.7)	0 (0.0)	2 (13.3)	2 (12.6)	1 (20.0)
Secondary vocational education	16 (34.8)	5 (33.3)	4 (26.7)	7 (43.8)	1 (20.0)
Applied university	16 (34.8)	6 (40.0)	5 (33.3)	5 (31.3)	1 (20.0)
University	10 (21.7)	4 (26.7)	4 (26.7)	2 (12.5)	2 (40.0)
Employment status^d^					
Student	3 (6.5)	1 (6.7)	2 (13.3)	0 (0.0)	1 (20.0)
Full-time work	11 (23.9)	4 (26.7)	1 (6.7)	6 (37.5)	1 (20.0)
Part-time work	7 (15.2)	0 (0.0)	4 (26.7)	3 (18.8)	1 (20.0)
Self-employed	2 (4.3)	0 (0.0)	0 (0.0)	1 (6.3)	0 (0.0)
Unemployed	1 (2.2)	0 (0.0)	0 (0.0)	1 (6.3)	0 (0.0)
Homemaker	1 (2.2)	1 (6.7)	0 (0.0)	0 (0.0)	0 (0.0)
On sick-leave/work disabled	23 (50.0)	9 (60.0)	7 (46.7)	6 (37.5)	2 (40.0)
Type of cancer					
(Low-grade) glioma	16 (34.7)	0 (0.0)	0 (0.0)	16 (100)	1 (20.0)
Sarcoma	7 (15.2)	6 (40.0)	1 (6.7)	0 (0.0)	1 (20.0)
Breast cancer	6 (13.0)	3 (20.0)	3 (20.0)	0 (0.0)	1 (20.0)
Lung cancer	6 (13.0)	0 (0.0)	6 (40.0)	0 (0.0)	1 (20.0)
Melanoma	3 (6.5)	0 (0.0)	3 (20.0)	0 (0.0)	0 (0.0)
Cervical cancer	2 (4.3)	2 (13.3)	0 (0.0)	0 (0.0)	0 (0.0)
Other^e^	6 (13.0)	4 (26.7)	2 (13.3)	0 (0.0)	1 (20.0)
Stage at time of interview^f^					
II	0 (30.4)	0 (0.0)	0 (0.0)	NA^f^	1 (20.0)
III	1 (3.3)	2 (13.3)	0 (0.0)	NA	0 (0.0)
IV	25 (83.3)	13 (86.7)	12 (80.0)	NA	2 (40.0)
N/A or unknown	4 (13.3)	0 (0.0)	3 (20.0)	NA	2 (40.0)
Current treatment^g^					
None/Active surveillance	16 (34.8)	4 (26.7)	1 (6.7)	11 (68.8)	2 (40.0)
Chemotherapy	14 (30.4)	9 (60.0)	2 (13.3)	4 (25.0)	1 (20.0)
Targeted therapy	10 (21.7)	0 (0.0)	10 (66.7)	0 (0.0)	1 (20.0)
Experimental therapy	3 (6.5)	0 (0.0)	2 (13.3)	1 (6.3)	0 (0.0)
Hormonal therapy	3 (6.5)	2 (1.3)	1 (6.7)	0 (0.0)	1 (20.0)
Immunotherapy	1 (2.2)	1 (6.7)	0 (0.0)	0 (0.0)	0 (0.0)
Radiotherapy	1 (2.2)	1 (6.7)	0 (0.0)	0 (0.0)	0 (0.0)
Comorbidity					
None	34 (74)	11 (73.3)	10 (66.7)	12 (75.0)	5 (100)
One	10 (21.7)	3 (20.0)	4 (26.7)	3 (18.8)	0 (0.0)
Two or more	2 (4.3)	1 (6.7)	0 (0.0)	1 (6.3)	0 (0.0)

^a^ Total N = total interviewed AYAs, AYAs participated in focus groups are not included. ^b^ Yes = Christian, Protestant, Islamic. ^c^ Other = living with brothers, living with parents and son, living with housemates. ^d^ Not equal to 100% since some participants were partly disabled and worked part-time for the other part. ^e^ Other = (1) traditional survivors: colon cancer, epithelioid hemangioendothelioma, neuro-endocrine tumor, ovarian cancer (2) new survivors: gastrointestinal stromal tumor. ^f^ Low-grade gliomas are grade 2 tumors according to WHO Classification of Tumors of the Central Nervous System. Two patients were initially diagnosed with a low-grade glioma, but at the time of the interview it was progressed to a high-grade glioma grade III/IV. ^g^ Not equal to 100% due to the combination of treatments.

**Figure 1:**
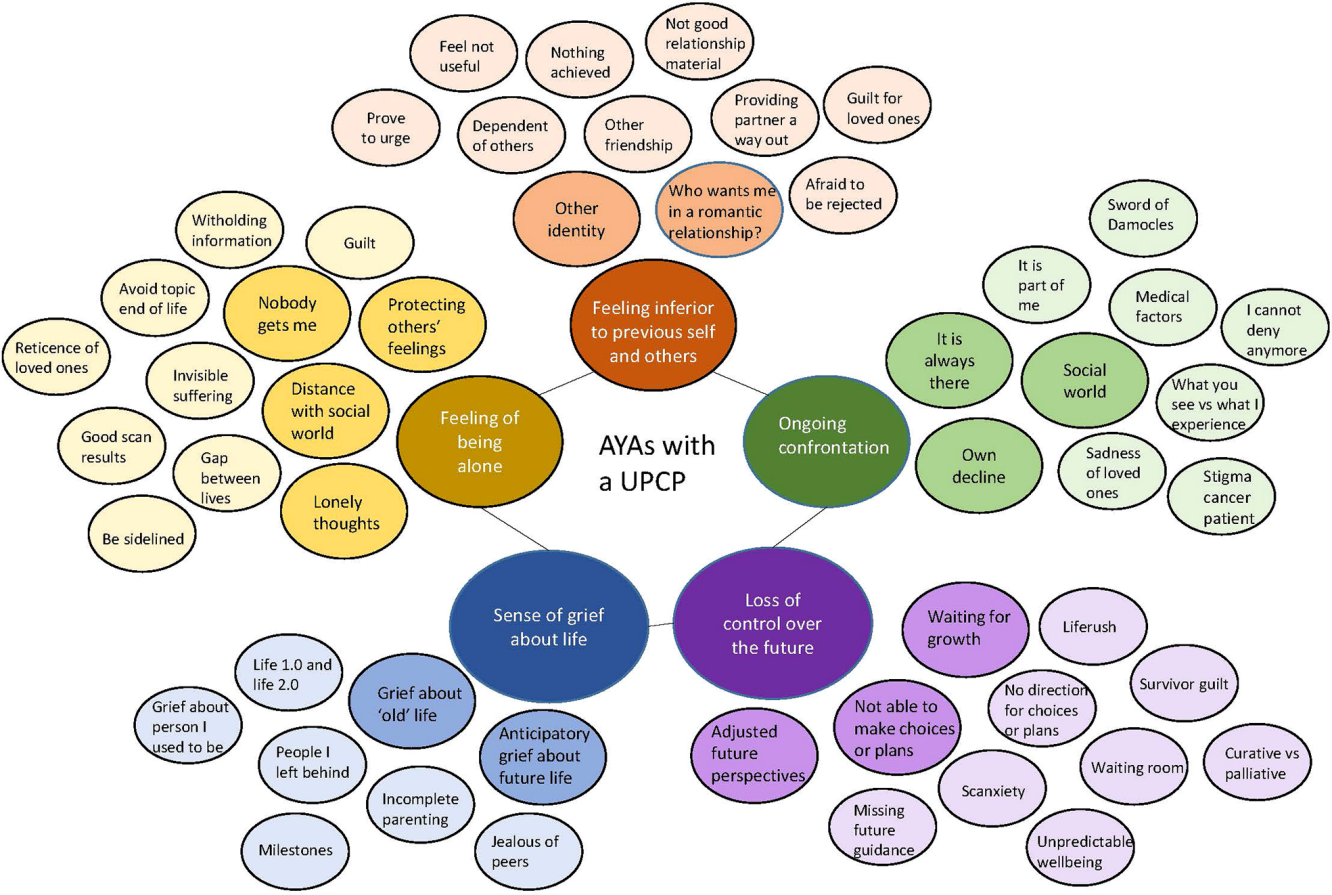
Visual overview of challenges for AYAs with UPCP experiences. The size of the inner circles represents the frequency of the reported challenge, e.g. the bottom two circles are the largest, which means that these challenges were experienced the most.

We identified five main themes in which we incorporated the influence of the COVID-19 pandemic according to the experiences of the AYAs with a UPCP. The themes, subthemes, and codes along with quotes are presented in **Table 3**. Numbers in parentheses denote the in-text reference for the quote in the table. A visual overview of the results is presented in **Figure 1**. **Table 4** provides a complete overview of differences between the AYA subgroups (**Table 4**).

**Table 3:** Coding hierarchy.

Theme	Sub-theme	Codes	In-text reference	Quotes
1. Feeling inferior to previous self and others	1.1 Other Identity	Feel not useful	1.1.1	I am just failing. [.] I think it is bad to say about myself that I feel inferior to others, but I do not feel equal to others. The fact that have a zero-hour contract for 2-3 days a week and even have to take days of because of hospital appointments. With a zero- hour contract you do not participate in the annual evaluation. I was called a ‘temporary employee’ in teams for over a year. All that doesn’t feel equal to me. (m- melanoma)
		Feel like I have nothing achieved	1.1.2	I want to become a professional. Because I notice so little progress and I already have issues exercising besides my six hours of work, is a scary thought for me. I worry that this is all I will be capable off and I will never make more than 1000 euros per month. That really sucks. (m- low-grade glioma)
		Prove to urge	1.1.3	During my probation period, it turned out that I had a brain tumor, so all my colleagues have a permanent contract and I do not because I am sick. So that already does not feel right, even though I am a really hard worker. (w- low-grade glioma)
		Other me, other friendship?	1.1.4	You make friends when you were a certain person. But I am not really that person anymore, I have changed quite a bit. And that makes me very insecure, will they still like me if I cannot do all of this anymore? (w- breast cancer
	1.2 Who wants me in a romantic relationship?	Not good relationship material	1.2.1	You wish someone the best, I don’t think I am the best right now. It feels like lying without me lying. And you know you are consciously are going to hurt someone because you are going to die. I don’t want someone on purpose and want to protect this person. (w- focus group)
		Afraid to be rejected	1.2.2	I just removed all dating apps. I thought just leave me alone. But in fact I really want nothing more than a relationship. But I am so afraid. in my head it is always: who wants me? (w- cervical cancer)
		Guilt for loved ones	1.2.3	He has a lot more to do, which is difficult for me. He has to take care for me as well as for the kids and I cannot do many household tasks. I think it is skew. I know there is no other way, but he has a job and when he comes home there is a new to do list for him … while I am at home. So he has a lot of responsibilities. It is more patient-caregiver kind of relationship and that is not how you want it. (w- breast cancer)
		Providing partner a way out	1.2.4	I recently presented him that he have the choice to leave me. I told him “you’re 35 years old. If we break up, than you have some extra years to find a new girlfriend and starting a family. In case I live for 5 more years, you are 40 and your change to start a family is relatively smaller. Do you realize that?” (w- osteosarcoma)
2. Feeling of being alone	2.1 Protecting others’ feelings	Withholding or weakening information	2.1.1	I actually already knew the scan results and I did not want to make them [parents] sad, so then I thought … well this is a good time to go alone because no one is really allowed to accompanied me [COVID-19 restrictions]. But yes, that was of course very stupid if you know that you will receive such a bad message. (w- low-grade glioma)
		Feelings of guilt	2.1.2	If you have received bad news and tell your family and see how much sad they are. seeing your grandparents cry for the first time makes you feel guilty about it and you don’t want to say or do not dare to say it next time. (m- focus group)
	2.2 Lonely thoughts	Avoid topic end of life	2.2.1	When I say it out loud, when I discuss it, it becomes reality. So, I dare not to say things out loud. (w- breast cancer)
		Reticence of loved ones	2.2.2	If I am worried or I am talking about. Or I heard a song on the radio and I think oh that is nice song for my funeral, I get a response like ‘You are not going to die at all’. (w- sarcoma))
	2.3 Nobody gets me	Invisible suffering	2.3.1	People do not understand. Because you just look normal on the outside. Yes, I mean when I go to a party there is no one who can see that I am sick on the outside. And they know it, but ‘your scans are stable, you can last for so many more years’. I am not someone who shouts it out loud and I also want to have a good time when I go out. But I do miss the understanding, especially from the people you would expect some understanding. It is very difficult for me. (w- melanoma)
		Good scan result	2.3.2	It feels like everyone is so relieved, like you had such good news so now we can put is behind us. Like if I have been cured, as if I am no longer a cancer patient anymore. While in fact I will never been cured and always remain a cancer patient. (w- focus group)
	2.4 Distance with family and friends	Be sidelined	2.4.1	I did not dare to tell you because you are struggling with your brain tumor and I call sick because I am not feeling well after giving birth. So you notice that people hesitate to involve me in their problems, because everything they experience is less important than my brain tumor. That is not how I see or feel it at all. It actually feels like you are no longer involved in things and that creates a certain distance. (w- low-grade glioma)
		Gap between lives	2.4.2	The whole evening they talked about weddings and the wish to have children, while I had a conversation with a cancer peer about funerals yesterday. I no longer feel the connection with my old friends, I cannot talk about the topics anymore because I am in a completely different life phase. It is confronting for me, because I cannot fantasize about these things. And this creates distance, because I cannot participate in their conversations. (w- focus group)
				My housemates are also going out, but they never ask me to go with them because they know that it is not possible. While they can just go out until 5 o’clock and I cannot, I sometimes feel alone. Because while the whole house is gone you already sleep in your room at 11 o’clock. It creates distance with your housemates. (m- focus group)
3. Ongoing confrontation	3.1 It is always there	It is part of me	3.1.1	You wake up with the cancer and you go to bed with it. It is part of my life; I am busy 24/7 with being sick, with the cancer, with peers and comparing with peers. (w- cervical cancer)
		Sword of Damocles	3.1.2	The cancer is on my mind at any time of the day, even when I go to the store with an action for shampoo. I really have trouble with that, I start to panic: ‘Do I need 3 shampoos? Am I still alive so I can by 3 shampoos?’ And I do not think my life will ever get back to normal. It is in the little things. (w- focus group)
		Medical factors	3.1.3	Going to a hospital is just confronting. Then you are dealing with the cancer: I am sick. And if the hospital appointments becomes more frequently, then it is more confronting for me. (w- lung cancer)
	3.2 Own decline	I cannot deny anymore	3.2.1	I always try to push it away, to do something else. But I notice that this is getting more and more difficult. [ … ] I keep getting worse with all kinds of things, with my memory and the fatigue. (m- low-grade glioma)
				I always think: this is a phase, later I will feel better. And my fear is that I am not feeling better anymore, so this is the best I am going to feel for now and in the future. (w- ovarian cancer)
		What you see vs what I experience	3.2.2	It is difficult to indicate my limits with my cognitive decline. For example, with work meetings, minutes have to be taken and then people expected that I can do that just like everyone else. But that is such a challenge for me. I can explain again that it hard for or I will just do it again. It forces me to either go over my limits again or tell everybody about my cognitive problems. (w- focus group)
	3.3 Social world	Stigma cancer patient	3.3.1	My mother was at my place again ‘I will do your laundry and then I will do the dishwasher and…’. And I think, I can do all of this myself and I am not totally incapable. People want to do this for you since they cannot do anything else, but I find it very difficult that people see me like this. (w- lung cancer)
				What I have often said to people is that I do not want to be seen as pathetic. And I do notice that people feel sorry for me, especially when I am in the hospital. I am always the youngest and people really looked at you wide-eyed, full of pity. That is difficult. (w- cervical cancer)
		Sadness of loved ones	3.3.2	It is a bit selfish, but it should relieve me. And if they cry very much or show how much it affects them, it just becomes a bigger thing in my head. (w- low-grade glioma)
4. Sense of grief about life	4.1 Grief about the ‘old’ life	Life 1.0 and life 2.0	4.1.1	I was difficult that also school was taken away from me. And that is not the only thing, because I am also very sporty and then was not able to exercise anymore. Just every time there are those little things you cannot do anymore. (w- lung cancer)
		Grief about the person I used to be	4.1.2	You have to say goodbye to so many things. From the fit [name], the fit mother. I saw myself skiing with the kids, running next to the bike, playing football and going to hockey. Then you have the work [name], the colleague [name], that is also part of my identity. And the energy [name]. They do not really exist anymore. That is very difficult sometimes and it makes me sad to say goodbye to those parts and process this loss. (w- breast cancer)
		The people I left behind	4.1.3	I know how much it will hurt the people I leave behind. It is a kind of guilt ‘oh god, what am I doing to these people’. It is not fair. Because we know that when someone dies, people are always left behind and can be very sad about it. The pain that will come when I am death that is what makes me sad and I do not want that. (w- leiomyosarcoma)
	4.2 Anticipatory grief about the life I did not/do not get	Milestones	4.2.1	I had an ideal image of my future and I had adjusted my life for the past 4-5 years in such a way that everything would fall into place. And just on that moment I got cancer. I wanted to go into the consultancy and that includes working weeks of 80-90 hours and I wanted to continue living abroad. Now, I am looking for a part-time position in the consultancy, had to take a step back for my top sport career and was moved in the Netherlands. (m- desmoplastic small round cell tumor)
		Incomplete parenting	4.2.2	The life lessons that you have experienced or learned as a person, that you can no longer pass them on to your child. That he has in the back of his mind: ‘yes mom, she always says this and that’. [ … ] And writing it down is different from conveying it yourself and that he knows me as a person. (w- leiomyosarcoma)
		Jealous of peers	4.2.3	I experience a kind of jealousy towards all people who can just do all the things I cannot. And I also experience jealousy towards my friends, because I see them working and starting an internship … and I would like to do that too. And I just find that very difficult. (w- lung cancer)
5. Loss of control over the future	5.1 Adjusted future perspectives	Missing future guidance	5.1.1	How I organize my life, what I have to take into account is always uncertain. It is like someone is constantly sawing my legs. It stops for a while, then there is a short break and one leg is shorter than the other, but you learn to live and deal with it. And then some saws again, it becomes a bit more skewed or with help a bit straighter again. But you know it never ends. So with a bad scan result, they are sawing again and you do not know how it ends. You did not know the impact from a bad scan result either. (w- breast cancer)
				As long as the treatment works, it is good. But I do not know how long that is and that is really hard for me. So I set goals like school and getting married to stick to them. And if that disappears for me, I do not see the point of either. It is not that I am done with life, but I need something to look forward to. (w- lung cancer)
		Scanxiety	5.1.2	It is just an update if your continue living. That is how it gets into my head. I did not work for a week because I was just completely exhausted from the weeks before the scan. I was ruminating and was exhausted because of the stress. (m- low-grade glioma)
	5.2 Not being able to make (life) choices or plans	No direction for choices or plans	5.2.1	I really need certain goals or things that I can plan. And the fact that you can only look 8-12 weeks ahead, that is not bad for a while but after 4 years it started to frustrate me. After 4 years I was no longer 28 but 32. So quite a lot has changed, but I am standing still or actually go back even further. I can plan a vacation and make small purchases such as a car. But big choices like starting a family is impossible, while our biological clock is ticking and you are confronting with everyone around you who will settle done. (m- melanoma)
				I am now in a position, I am 28 years old and my study is still 2 years. And I really have that frustrating feeling of. okay but what if I only have a year and a half left? Then I spend a year and a half to my education and then I die. And I would find that very frustrating. So I really want to spend the time I have on fun things, on things I really want to do. (w- breast cancer)
		Life rush	5.2.2	I put a little pressure on myself [ … ] A bit of an adult life. I want to do or achieve what my parents have. I think I must have taken everything out. (w- low-grade glioma)
		Unpredictable wellbeing	5.2.3	It is also just hard to plan dates with friends. My friend wants to make an appointment to play a board game or something on Saturday but I had to cancel it. Then he has to keep the next Saturday free and I do not want him to keep his Saturday free if I do not know if I am going to make it. So it makes it more difficult to keep in touch. (m- low-grade glioma)
	5.3 Waiting for growth	Sitting in a waiting room	5.3.1	You are in the waiting room, but you do not know when it is your turn. And there is no indication that you have got 10 minutes left. There is not ten or twenty minute leeway, you do not know what is about to happen. So it is just a matter of waiting. (w- leiomyosarcoma)
		Survivorship guilt	5.3.2	I met a girl during cancer treatment and we became friends. She had a semi- good prognosis and I did not, however, she died and I am still here. I felt guilty regarding her family assuming they did not want to see me since I am still alive and their daughter is not anymore.” (w- focus group)
		Curative versus palliative	5.3.3	[ … ] then at least you are doing it for something. There is a dot somewhere that you live for, like ‘Well, I have to do this. I am almost out of strength but I am not giving up because eventually I will be better and then I can live to 100. Then I can start planning my future and get on with my life.’ And now you have a lot of trouble and you are almost at that dot at the horizon but that point keeps disappearing, it won’t come. You are fighting something you can never win, you know you are going to lose in the end. (w- melanoma)

m, man; w, woman.

**Table 4:** Differences in experienced challenges between AYA subgroups.

Themes, sub-themes, codes	Less often reported by	More often reported by
1. Feeling inferior to previous self and others	New survivors	
1.1 Feeling not useful		Low-grade glioma survivors
2. Feeling sense of loneliness	Men	
2.3 Nobody gets me	Traditional survivors	
3. Ongoing confrontation	Traditional survivors	
3.1.3 Medical factors		New survivors
3.2.1 I cannot deny anymore	Traditional survivors	
5. Loss of control over the future	-	-
5.1.2 Scanxiety	Traditional survivors	
5.2.1 No direction for choices of plans	Low-grade glioma patients	

### Theme 1. Feeling inferior to previous self and others

#### 1.1 Other identity

The physical, mental, and psychosocial (long-term) challenges that come together with an advanced cancer diagnosis require from AYAs to find a new balance in life and ask for an adjusted identity. AYAs with a UPCP relate their identity specifically to work or education. Adjustments to work life or education (e.g. stop working, working fewer hours) create feelings in AYAs as if they are not useful, like they achieved nothing or as if they lost the connection to society, leading to diminished self-esteem (1.1.1/1.1.2). Adaptions in work life were in general more present due to COVID-19 and the feelings as described above were especially the case in AYAs with a low-grade glioma. Since AYAs did not want to be identified as patients; some of them have the urge to prove themselves at work, especially when they have the idea to be treated differently by colleagues or employer due to their cancer (1.1.3). Younger patients wanted to identify themselves as an excellent patient, who is able to finish their study to show that they are no quitters and stay part of the society.

In addition, the uncertain future leads some AYAs to anxiety around managing a work task or returning to work due to concerns that they might not have the physical or cognitive ability to do so. Additionally, AYAs relate their identity to their role as a friend and parent (1.1.4). All together, these experiences make AYAs feel inferior to their previous self and others, which was mostly the case in AYAs with low-grade glioma and traditional survivors.

#### 1.2 Who wants a romantic relationship with me?

AYAs experience challenges forming new romantic relationships as they are feeling inferior to the other and feel guilty to hurt another because of their cancer and potential premature death (1.2.1). Afraid to be rejected because of their cancer diagnosis, some AYAs are struggling with finding the right moment to share their cancer story while dating and others totally avoid dating (1.2.2). AYAs with a relationship experienced a sense of guilt for their loved ones because of the extra practical burden on the shoulders of their partner, the tension of the situation, and the perceived inequality as they feel they cannot give their partner what he or she deserves (1.2.3). Because of the uncertain prognosis in a life stage full of milestones, existential questions emerged, like ‘Is it fair that he cannot become a father because of me?’ AYAs reconsider their relationship and some of them ask their partners whether they want to continue their relationship to provide them with a way out (1.2.4).

### Theme 2. Feeling of being alone

#### 2.1 Protecting others’ feelings

AYAs do have a strong need to protect feelings of loved ones by not sharing everything, not telling all the details, or toning down bad news (2.1.1). AYAs do not want to be a burden and described feelings of guilt when confronted with the sadness of their loved ones, especially parents who are more often quick to switch to panic mode. Even though they know they cannot do anything about it, they still feel like they are the cause of all problems (2.1.2). Not sharing everything results in feelings of being alone.

#### 2.2 Lonely thoughts

Some AYAs are not able to discuss everything about the cancer with their loved ones, especially about end of life. As some AYAs avoid specific topics themselves (2.2.1), others experience reticence by their loved ones. This reticence of their loved ones is noticeable by the intention to deviate from the subject or by downplaying the severity with comments like ‘you are not going to die’ (2.2.2). AYAs explain this by the fact that they are a few steps further in this thinking process than their loved ones. Some AYAs express not feeling supported by their partner, as the partner is coping completely differently with the situation or is not able to talk about the cancer. AYAs also do not want to be a burden when initiating a subject. All of this result in these lonely thoughts about cancer, and especially death and dying remain in the mind of the AYAs themselves. It is remarkable that in some cases the COVID-19 pandemic was the reason to discuss this topic.

#### 2.3 Nobody gets me

For AYAs with a UPCP it feels like nobody really understands what they are going through. The complexity of their (rare) tumor combined with the invisibility of living with an uncertain and poor cancer prognosis and the lack of peers in comparable situations results in misunderstanding and a lack of empathy from others. Good days without physical issues can be interpreted by their environment as if the AYA is doing really well, while bad days and mental issues remain unspoken (2.3.1). The biggest misunderstanding is related to good scan results, in which it feels like everyone is relieved, the panic is gone, and the cancer can move to the sideline, while the AYAs themselves do not feel that kind of relief since there is still a threat of death and the stress of a new scan in a short time (2.3.2). This response from the environment results in AYAs struggling to talk about cancer-related issues and some even distance themselves from their family and friends.

#### 2.4 Increasing distance to the social network/environment

The social environment wants to protect the AYA by not sharing everything, bringing their issues in perspective (‘my issue is nothing in comparison with your cancer’), and asking others about the AYA instead of talking to the AYA themselves. The environment makes decisions about what the AYA can and cannot handle, which feels like the AYA is moved to the sideline and makes AYAs think about the value of their relationships (2.4.1). Additionally, the significant differences between the life of the AYAs and their peers result in an ever-increasing distance and confrontation since most of the time they cannot join in the normal AYA age-related conversations about parties, weddings, career, and parenthood, causing social withdrawal in some AYAs (2.4.2).

It is remarkable that feeling alone is more reported by women than men.

### Theme 3. Ongoing confrontation

#### 3.1. It is always there

AYAs report their cancer is 24/7 on their mind; it is part of their daily life (3.1.1). Even when the cancer is stable and people expect that these thoughts move to the background, AYAs express that they always think of the sword of Damocles above their head because of their shortened life expectancy (3.1.2). For the new AYA survivors, the cancer is usually more on the background. However, they experience their fluctuating physical or mental well-being and the hospital appointments as confronting factors, switching the cancer to be on the foreground (3.1.3).

#### 3.2 Own physical and cognitive decline

When denial of the cognitive and physical issues is not possible any longer, AYAs are confronted with their own decline (3.2.1). This is mainly the case by AYAs with low-grade glioma followed by new survivors with an uncertain prognosis. For some AYAs their decline feels like a sacrifice they have to make repeatedly, which impacts every aspect of their life. Others experience anxiety of how this (unknown) cognitive decline ends. In some cases, AYAs are capable to compensate their decline by using mnemonics and other tricks. This results in the situation that their decline is not always noticed by their environment, with the risk that too much is expected from the AYA (3.2.2). Although, the decline is not always remarkable, AYAs themselves are everyday conscious of how it was before and how it is now.

#### 3.3 Environment

The wish of the AYA to be normal is in contrast with the cancer stigma in the society and the attitudes of the environment towards AYAs with a UPCP. Difficulties with a driving license due to the epilepsy (in low-grade glioma), challenges with buying a house, and not getting a permanent contract are issues contributing to a confrontation that the AYA is no longer participating fully in society. The AYA and the cancer are also often the center of attention of their social network. Concerns of their loved ones are mostly expressed in searching for more contact, taking over tasks and making decisions for the AYA which make the AYA feel more dependent. AYAs are also experiencing that the environment feels sorry for them, which makes the AYA actually feel like a patient (3.3.1). Additionally, the sad emotions of their loved ones make AYAs realize that their situation is indeed serious. This sadness is also too much to handle since they have enough of their own sorrow. Instead of providing relief by sharing thoughts, it provides a conformation of the concerns the AYA has, resulting in protecting themselves by not sharing everything with their loved ones (3.3.2).

### Theme 4. Sense of grief about life

#### 4.1 Grief about the old life

AYAs with a UPCP experience grief about their pre-diagnosis life since psychosocial, cognitive, and physical issues cause limitations in all areas of life. Many aspects of their lives have stopped because the cancer or adaptions have been made regarding study, work, and relationships (4.1.1). The whimsical pattern of this uncertain and poor cancer prognosis ensures that people continue to deteriorate and therefore have to give up more and more throughout their lives. This repeated loss experience creates grief about the person they used to be and creates questions about their own identity (4.1.2). Although, this group has no other option than dealing with this grief, most of them cannot accept the changes and have the wish to go back to their old life. Saying goodbye to the old life also includes saying goodbye to their loved ones. Leaving their loved ones behind because of the fact that you will die prematurely is the most painful part for AYAs with a UPCP. It felt like they are going to hurt their loved ones by abandoning them, and not being able to provide comfort makes it emotionally very hard (4.1.3). AYAs experience worries about how their loved ones are coping after their death and some are even afraid that they have not done enough to leave their loved ones behind in the best possible way (e.g. not enough savings). In some cases, these worries resulted in an extreme need of control and others keep people at a distance so they do not have to hurt them either.

#### 4.2 Anticipatory grief for the life I did not/do not get

Many AYAs are working hard to accomplish their future goals. Because of the cancer and its consequences, it is the question whether these goals and other milestones can be achieved including lost parenthood, not being able to buy a house, and giving up your dream job (4.2.1). This asks for a new vision of the future, which often goes hand in hand with ethical dilemmas (e.g. is it ethical to start a family in my situation)?. Furthermore, young parents experience anticipatory grief for missing many milestones from their children, not knowing what kind of person their child will become, and not being able to provide their children with life lessons (4.2.2). Seeing your peers living their life and achieving future goals is experienced as an extra confrontation by AYAs. This causes feelings of jealousy, especially when peers are doing activities AYAs are not capable of anymore (4.2.3). The feeling that they will never achieve the same level as their peers feels unfair and for some AYAs, resulting in the struggle of having trouble to be happy for a peer when achieving a life event.

### Theme 5. Loss of control over the future

#### 5.1 Adjusted future perspectives

Majority of the AYAs with a UPCP are forced to adjust their ideas about the future. Instead of most of their peers, AYAs do not think and plan long term and some do not even dare to dream of a future. Most of them are focusing on the current situation and planning a week or a few weeks ahead. Others are planning for a year, hold on to the life expectancy range they received by their specialist, or focus on a goal like graduating or marriage so they have something to live for. Not having a future perspective or even a bit of control and not even knowing what you live for are experienced as the hardest part by these AYAs and asks for an enormous flexibility in their mindsets. This flexibility is being required since scan results and treatment can change their daily life and future direction (5.1.1). Therefore, many AYAs plan their future from scan result to scan result as this determines the rest of their life, which comes with enormous stress and sometimes even impacts their functioning and wellbeing for a period of time (5.1.2). It is remarkable that this stress is not mentioned by many AYAs in the traditional survivor subgroup. Some AYAs experienced their loss of control over the future more easily during the COVID-19 pandemic, since everybody in the Netherlands was forced to adjust their future plans and experienced some uncertainty.

#### 5.2 Not being able to make choices or plans

Most of the new and traditional AYA survivors report that being young and not having a clear future perspective makes it hard to make (life) choices, because a good rationale to base choices on is lacking. “What is a good choice in this situation, what is it worth?” Topics like buying a house, starting a study, quitting your job, and starting a hobby are difficult if nobody can tell you how long you have left to live. The wish to become a parent is an extra ethical discussion (5.2.1). Mainly new and traditional AYA survivors express that they feel out of control and that they are constantly wondering whether they are making the right choices. Several AYAs experience suicidal thoughts, as this will end the uncertainty. Some are struggling with a sense of rush of life; they want to do as much as possible because death can suddenly appear. This creates a lot of pressure, little time for relaxation, and the idea that relaxing is a waste of your time (5.2.2). An extra challenge is the unpredictability these AYAs experience in daily life according to their physical and mental health. Everything they plan is with reservation of their wellbeing, which they have no control of and results often in canceling plans and being disappointed (5.2.3). For some it feels like they are not in the lead of their own lives. AYAs express that during the COVID-19 pandemic, they experience less fear of missing out since everyone was forced to cancel their plans.

#### 5.3 Waiting for growth

AYAs explain that whatever they do the only certainty is that they will eventually die prematurely of cancer. It is hard for them to know that no matter what they do, no matter how tough they are, or how hard they work, the outcome remains the same (5.31). Waiting for growth is for AYAs in the focus group associated with experiencing survivors’ guilt, which implies struggling from simply being alive, especially when many of their peers from the cancer community may not have survived (5.3.2). Sometimes the motivation to get out of bed and undergo a new treatment is lacking since there is no hope of curation. Except for extra time with hopefully good quality of life and time with their loved ones, a clear and healthy future is missing (5.3.3).

## Discussion

With this study we are the first to describe the psychosocial challenges in daily life specifically for the group of AYAs with a UPCP. In collaboration with AYA research partners, we identified four main challenges with an impact on all areas of life: feeling inferior to previous self and others, feeling of being alone, ongoing confrontation, grief about life, and loss of control over the future. Although all of the challenges were identified in the different AYA subgroups (traditional survivors, new survivors, and low-grade glioma survivors), the perceived intensity of the challenges differed slightly between the subgroups. The experienced psychosocial challenges seem not to differ by tumor type or stage of treatment other than those included in the AYA subgroups.

The first challenge, *feeling inferior to previous self and others*, shows that AYAs with a UPCP felt useless since they were not being able to perform their work in the same way as they did before while work was part of their identity and reminded them of their “normal” life. It is remarkable that this was mainly reported by AYAs with a low-grade glioma. A possible explanation could be the dissonance between their physical and cognitive functioning, which makes it even harder to accept the adjustments in work since some of them still had the physical capacity to work but suffered from cognitive dysfunction. Besides this loss of work identity, our results show that AYAs do not see themselves as a good romantic partner since they cannot offer what a healthy peer can offer. In line with previous research, we found that AYAs with UPCP have to deal with many loss experiences, regarding everything they could do in their pre-diagnosis life as well as the person they used to be. They also experienced the loss of their future dreams or lost opportunities to participate in developmental milestones like becoming a parent, which affected their sense of purpose (7, 10, 13).

Related to the identified challenges of *grief about life*, AYAs seems to feel broken, which is an accumulation of three potential causes (1): the differences between the pre-diagnosis and current situations (2), loss of their old life and future life, and (3) the internalized societal thoughts of the AYAs according to their impairment. According to the ‘contingent hope theory’, periods of feeling broken and experiencing loss may accumulate to a point where AYAs lose orientation to almost every facet of their pre-cancer identity, which is called disorienting grief (7, 10). In our study, disorienting grief was reported by several AYAs. It may seem possible that the other AYAs found themselves in other stages of the theory in which they were focusing on reframing mindsets and daily priorities to balance their experiences of loss or identity reconciliation. Currin-McCulloch et al. portrayed that hope plays an important role in motivating AYAs through disorienting grief toward finding a new balance; however, situational or medical changes can start the process of loss and feeling broken again (7). Additionally, AYAs with a UPCP reported experiencing *ongoing confrontation* since their poor prognosis is always on their mind or medical factors like hospital appointments confronted them on a regular basis. In line with previous research, we found that being conscious about their physical decline and the fact that people treat you differently are additional confronting factors for this patient group (15, 26).

Our results showed that almost nobody truly understood the unique situation of AYAs with a UPCP, which resulted in their *sense of feeling alone.* Studies by Currin-McCulloch and Knox suggest that this might be explained by the mismatch with curative AYAs since they can return to their normal life with future life goals, which was also reported by the AYAs in our study (7, 10, 12). They felt alone in their thoughts about death and dying, felt sidelined, or even took some distance on their own since they wanted to protect their loved ones or do not fit in the old life of their peers anymore. It seems possible that men do not feel the same social needs as women, since these feelings of loneliness were mainly reported by AYA women. Earlier research suggest that men use more problem-focused coping strategies (active efforts to eliminate the stressor) instead of emotion-focused coping (changing the emotional response to stressor), reported information as emotional support, and were possibly reluctant to admit needing emotional support in contrast with women who seek emotional support from numerous sources (27, 28).

All these loss experiences form an extreme burden. On top of that, AYAs with a UPCP are dealing with these losses while not experiencing any *control over their future life* due to medical uncertainties (13). This constantly asks for a flexible attitude to find a new balance in what they are capable of (and what not anymore), who they are, and how their future looks like. Adjusting their future by planning day by day or from scan to scan goes along with not being able to make long-term plans or choices due to their uncertain prognosis. Shiling and colleagues suggest that living with uncertainty is difficult in older melanoma patients, but probably even more difficult in young cancer patients (29), since AYAs have to make many choices about their future life (e.g. should I start a new study, should I start a family) without any hold on or end point to help them making the right choices. The extra difficulty of dealing with advanced cancer in a vulnerable life stage of AYAs is supported by research of Lie et al. suggesting that AYAs have fewer life and coping experiences (26). It is interesting that AYAs with a low-grade glioma did not frequently report issues around a lack of direction for choices or plans due to the uncertain future. It seems that the majority of these patients already have made choices in life, since just slightly more of these AYAs are in a relationship and are a parent, which may provide some future direction. A relatively longer life expectancy and multiple treatment options may offer another explanation. In some cases, loss of control over the future increases the need to live a meaningful life and rush to accomplish milestones regardless of the time they have left (12). For other AYAs who are not experiencing a rush of life, missing that dot on the horizon sometimes feels like they have nothing to look forward to.

### Limitations

While this qualitative study included relatively large numbers of AYAs with a UPCP, the representation of ethnic minority groups was limited, which makes it hard to generalize the conclusions to this entire population. Future research should focus specifically on the challenges of the ethnic minority groups in order to provide culturally appropriate care. Additionally, we were not able to include AYAs below the age of 23 years, and the man/woman ratio was skewed. This slightly limits the generalizability of our results. The data of this study are based on a single interview with each AYA to examine the primary experiences, while it is possible that the challenges experienced by AYAs with a UPCP are changing over time. We aimed to cover the differences in challenges over time by including a heterogeneous group of AYAs with a UPCP with diversity in diagnosis, years since diagnosis, and treatment. Additionally, we asked the respondents if they assume whether their answers would change if they were in a different (disease) phase, to which half of them replied with yes. Longitudinal future research should get a better understanding of the age-specific risk factors that contribute to the development, maintenance, or fluctuation over time of the challenges we identified in this study. This future research may provide an explanation for the differences we found between the three AYA subgroups.

### Clinical implications

AYAs with a UPCP get a lot of respect from social media, family, or medical staff on how well they handle their situation, yet they do experience daily challenges. This study highlighted the complexities of AYAs living with a UPCP. These challenges appeared to be specific for AYAs with a UPCP but are not always openly shared by the AYA or are simply not visible for everyone. This calls for healthcare professionals to ask the right questions. We can learn from the example of the Dutch AYA Care Network, which developed an AYA topic list to assist healthcare professionals to examine the typical care needs of AYAs (e.g. fertility, education and work, death, meaning of life) and also provide some interventions to meet their needs (e.g. refer timely to palliative team) (30). Although some topics match with the challenges reported in this study, the current topic list is lacking specific issues or follow-up questions regarding AYAs with a UPCP (e.g. feeling an inferior identity, sense of loneliness – protecting loved ones, loss of control – future plans). Therefore, we recommend adding the identified themes of this study as topics and the sub-themes as follow-up questions to focus on the loss experience of this group. Since AYAs with a UPCP experienced a high level of anxiety for scan results (‘scanxiety’), and some of them plan their lives around the returning scan results, it is valuable to look at the practical considerations of the frequency of the scans and the wait time between scans and results in relation to the psychological burden for the patients (31). Remarkably, anxiety regarding scans was less present in the group of traditional survivors, possibly explained by the fact that this group lives by their chemo-scheme instead of scan by scan.

To minimize social isolation and lonely grief, we should focus on peer-support initiatives. Earlier research found that social support was associated with better psychological and existential quality of life and less severe grief in young adults with advanced cancer (32). Trevino and colleagues suggest that providing context to discuss their experiences and meeting their practical needs may be the most effective way of social support (32). However, our study results as well as previous research suggest that AYAs with a UPCP are not able to relate with AYAs with curable cancers (7, 10, 12). It would be beneficial to start peer support groups exclusively for AYAs with a UPCP. Since social support by family and friends cannot be underestimated, AYAs with a UPCP may need assistance balancing the need to be supported by sharing information and withholding of information in relation to their desire to protect their loved ones (15). Additionally, men in this study did not seem to suffer from social isolation; however, we do not know if they are reluctant to seek and accept support. Therefore, gender roles should be taken into account when providing support to make sure than men’s needs are not minimized (27). How to provide support to AYA men could be a topic of future research. Furthermore, many respondents commented on how the study interview itself was perceived as helpful, which possibly argues for embedding time to listen and acknowledge the issues of this unique group in daily clinical care programs.

Since the situation and some of the issues this group encounters will never change (e.g. adjustments to work, premature death) and the thoughts and moods AYAs with a UPCP experience may be accurate appraisals of their present circumstances, we have to focus on the things we can change. In AYAs with a UPCP, this means focusing on the relationship with negative thoughts and emotions rather than changing the thoughts and emotions themselves. In this unique patient group, it seems valuable to focus on interventions like Acceptance and Commitment Therapy (ACT), an evidence-based therapy for people learning to deal better with their unpleasant feelings, taking a step away from negative feelings and focusing on the important things in life (33). Although ACT may be beneficial for a range of psychological disorders and some chronic illnesses and conditions, ACT studies in (advanced) cancer are limited. However, promising pilot data is present (33). Earlier systematic reviews cautiously suggest that ACT may be a beneficial way to improve depressive symptoms, anxiety, psychological distress, psychological flexibility, and aspects of health-related quality of life in adult patients with advanced cancer (34, 35). Future research should focus especially on the coping of AYAs with a UPCP to gain better understanding of how this group can cope with the daily challenges related to their UPCP and how to support them to improve their quality of life. Results show that living longer can also coexist with survivor guilt when identifying with someone who was going through a similar experience and has died. It is an emotional response of guilt connected to a sense of helplessness, powerlessness, and a deep sense of injustice. Since we are used to focus on the positive effects of surviving longer, it is import to be aware of the possibility of survivor guilt in AYAs especially when they are having peer contact (36). Future research should focus on survivor guilt in AYAs more specifically with attention for the risk factors and therapeutic interventions (e.g. cognitive behavioral therapy).

Lastly, this study is far ahead in involving patients as AYA research partners. This collaboration provides more appropriate and relevant research regarding AYAs with a UPCP and contributed to a better translation to suggestions for clinical practice. Furthermore, AYAs were proud to contribute, appreciated the feeling of being seen to be of added value, and received support from the contact moment with other AYA research partners. More details about collaboration with the AYA research partners and its benefits will be presented in a separate article.

## Conclusion

This study identified unique challenges of AYAs living with a UPCP, suggesting that the difficulties are particularly associated to their sense of altered identity, their position in the social network, and their future uncertainties. Since specific care for this patient group is net yet embedded in the healthcare system, this study highlights the importance to recognize and acknowledge the unique challenges of AYAs with a UPCP in AYA care programs. Taking into account the differences in perceived challenges between AYA subgroups and gender, it is essential to focus on personalized care. To provide age-specific care, we recommend the implementation of acceptance and commitment therapy and AYA peer support to support AYAs to live well with their UPCP.

## Data availability statement

The raw data supporting the conclusions of this article will be made available by the authors, without undue reservation.

## Ethics statement

The studies involving human participants were reviewed and approved by Institutional Review Board of The Netherlands Cancer Institute (IRBd20-205). The patients/participants provided their written informed consent to participate in this study.

## Author contributions

OH and WG conceptualized the study and acquired funding. VB, SiF, SuF, NH, MN, AD, WG, and OH developed methodology. VB, MvdB, LD, RL, JT, AC, MK, MB, AdL, and WG contributed to patient recruitment. VB performed interviews and analysis with help of MR. SiF, SuF, NH, MN, AD, WG, and OH were involved in analysis discussions. The original draft was prepared by VB, WG, and OH. All authors reviewed and edited the manuscript. All authors agreed to the published version of the manuscript.

## Funding

OH and VB are supported by a grant from the Netherlands Organization for Scientific Research (grant number VIDI198.007).

## Conflict of interest

The authors declare that the research was conducted in the absence of any commercial or financial relationships that could be construed as a potential conflict of interest.

## Publisher’s note

All claims expressed in this article are solely those of the authors and do not necessarily represent those of their affiliated organizations, or those of the publisher, the editors and the reviewers. Any product that may be evaluated in this article, or claim that may be made by its manufacturer, is not guaranteed or endorsed by the publisher.
